# Is it possible to identify cases of coronary artery bypass graft postoperative surgical site infection accurately from claims data?

**DOI:** 10.1186/1472-6947-14-42

**Published:** 2014-05-29

**Authors:** Tsung-Hsien Yu, Yu-Chang Hou, Kuan-Chia Lin, Kuo-Piao Chung

**Affiliations:** 1 Institute of Healthcare Policy and Management, National Taiwan University, Taipei, Taiwan; 2 Master Degree Program of Public Health, National Taiwan University, Taipei, Taiwan; 3 Department of Chinese Medicine, Tao-Yuan General Hospital, Ministry of Health and Welfare, Taoyuan, Taiwan; 4 Department of Bioscience Technology, Chuan-Yuan Christian University, Taoyuan, Taiwan; 5 Department of Health Care and Management, National Taipei University of Nursing and Health Sciences, Taipei, Taiwan

**Keywords:** Administrative data, Identification model, CABG, Surgical site infection, Decision tree, Classification and regression tree

## Abstract

**Background:**

Claims data has usually been used in recent studies to identify cases of healthcare-associated infection. However, several studies have indicated that the ICD-9-CM codes might be inappropriate for identifying such cases from claims data; therefore, several researchers developed alternative identification models to correctly identify more cases from claims data. The purpose of this study was to investigate three common approaches to develop alternative models for the identification of cases of coronary artery bypass graft (CABG) surgical site infection, and to compare the performance between these models and the ICD-9-CM model.

**Methods:**

The 2005–2008 National Health Insurance claims data and healthcare-associated infection surveillance data from two medical centers were used in this study for model development and model verification. In addition to the use of ICD-9-CM codes, this study also used classification algorithms, a multivariable regression model, and a decision tree model in the development of alternative identification models. In the classification algorithms, we defined three levels (strict, moderate, and loose) of the criteria in terms of their strictness. Sensitivity, specificity, positive predictive value, negative predictive value, and accuracy were used to evaluate the performance of each model.

**Results:**

The ICD-9-CM-based model showed good specificity and negative predictive value, but sensitivity and positive predictive value were poor. Performances of the other models were varied, except for negative predictive value. Among the models, the performance of the decision tree model was excellent, especially in terms of positive predictive value.

**Conclusion:**

The accuracy of identification of cases of CABG surgical site infection is an important issue in claims data. Use of the decision tree model to identify such cases can improve the accuracy of patient-level outcome research. This model should be considered when performing future research using claims data.

## Background

Healthcare-associated infection has become an important issue in the past decades, and large studies have been implemented using claims data [1-3]. In contrast to surveillance data, the use of administrative data can increase sample sizes due to reduced labor intensity, and it makes multi-institutional studies easier to implement [4]. The use of claims data can also facilitate the efficiency and standardization of case identifications [1].

Researchers have identified cases of infection in claims data through use of the International Classification of Diseases, 9th Revision, Clinical Modification (hereafter referred to as ICD-9-CM-based model). However, several studies have indicated that this model might be inappropriate for identifying such cases in claims data [5-8], primarily due to insufficient code lists or distortion by the payment scheme and other factors [1,7,9,10], and these problems might also affect the accuracy of studies, especially in patient-level studies [6,11]. The use of surrogate markers or development of identification models has been popular in the identification of cases of healthcare-associated infection since 2000. Current studies usually adopted one of the below three approaches. The first approach is the classification algorithms, in which unweighted algorithm with consecutive dichotomous decision steps is used to enable screening of high-risk patients based on limited number of electronic data sources [12]. Researchers first define several criteria, and if a case satisfies (or exceeds) a specific number of criteria (such as antibiotics utilization), then it will be identified as a case of infection. For example, Lee et al. defined four criteria for identifying cases of infection in gastrectomy patients, and if any criterion was met, a case of infection was identified [4]. The second approach is the multivariable regression model. After manual review of high-risk patients, this model incorporates electronic data sources into a weighted regression or prediction model to divide patients into low- and high-risk groups [12]. Researchers usually calculate a risk score based on microbiology indicators of each case and identify cases of infection by an optimal cut-off point. For example, Fujii et al. used blood culture and medical use indicators to calculate a risk score by the logistic regression model, and the optimal cutoff point was determined according to the resulting receiver operating characteristic (ROC) curve [13].

The third is the data mining approach (e.g. decision tree/classification and regression tree, artificial neural network, etc.), which is widely used for predicting and classifying objects, especially in the fields of information technology, operational research, and advanced biostatistics. The characteristics of data mining approach are: automatic discovery of patterns, prediction of likely outcomes, creation of actionable information, and focus on large data sets and databases. However, it is rarely used in the field of healthcare-associated infection [14,15]. Regardless of the approach used, the sensitivity, specificity, positive predictive value and negative predictive value were usually adopted to evaluate the performance of the model, and it is found that alternative identification approaches all had model performance better than that with the ICD-9-CM-based model.

Although research quality can be improved by improving the accuracy of identification of infection cases, there is no study, to our knowledge, that has compared these three approaches, and no study has differentiated which approach is the best. In addition, the best approach for the Taiwan National Health Insurance Database is unknown. Therefore, the purpose of this study was to use these approaches to develop alternative models for identifying cases of coronary artery bypass grafting (CABG) surgical site infection; all approaches (ICD-9-CM-based model and the alternative approaches) will be compared to surveillance by infection control personnel, and then the performance of the ICD-9-CM-based model will be compared to the other methods. Furthermore, model verification was also implemented in this study.

## Methods

This retrospective study used data from coronary artery disease patients admitted to two medical centers in Taiwan for CABG surgery. The performance of the ICD-9-CM-based model and five alternative models was compared in this study.

### Data sources

The 2005–2008 National Health Insurance claims data and healthcare-associated infection surveillance data from two medical centers were used in this study. There were 1,017 CABG surgeries performed at medical center A and 845 performed at medical center B, with 24 surgical site infections (SSIs) occurring at medical center A and 17 occurring at medical center B.

The dataset from medical center A was used for data training (model development), and the dataset from medical center B was used for model verification. The National Health Insurance claims data is a de-identified secondary database containing patient-level demographic and administrative information. Treatment items were aggregated and were without time-related information. For example, if a patient received 1 ml cefazolin before surgery, and another 1 ml during surgery, and then 1 ml after surgery, it will be presented as 3 ml of cefazolin during the hospitalization.

### Ethical statement

The protocol for this study was approved by the Institutional Review Board of the National Taiwan University Hospital (protocol #201001027R). The dataset we used in this study was the secondary data, all information was de-identified by data owners. Inform consent was not necessary in current study.

The protocol for this study was approved by the Institutional Review Board of the National Taiwan University Hospital (protocol #201001027R).

### Exclusion criteria

Patients were excluded from analysis if they were: (1) aged <20 years, (2) had postoperative surgical site ***infection*** due to prior operation, and (3) mortality after surgery in hospital.

### SSI case identification based on ICD-9-CM (ICD-9-CM-based model)

SSI case identification based on the ICD-9-CM was divided into two categories: index hospitalization events and post-discharge events (SSIs that occur within 1 year after discharge and require readmission to a hospital and/or the use of ambulatory services). The ICD-9-CM codes for hospitalization events are 996.03, 996.61, 996.72, and 998.5. The ICD-9-CM codes for ambulatory services events are 038.0-038.4, 038.8, 038.9, 682.6, 682.9, 780.6, 790.7, 875.0, 875.1, 891.0, 891.1, 996.03, 996.61, 996.72, 998.3, and 998.5. Following Wu et al., this study adopted the secondary ICD-9-CM diagnosis codes for index hospitalization events, and the primary and secondary diagnosis codes for post-discharge events as criteria for SSI to avoid cases in which infection developed prior to hospitalization [16].

### SSI case identification based on surrogate indicators

After referring to the literature [1,17] and conferring with infectious disease specialists, we determined the criteria used by the alternative models to be the following: type of antibiotics, doses of antibiotics, doses of cefazolin, use of second-line antibiotics (see Table 1), length of hospital stay, and number of vessels obstructed.

**Table 1:** Second-line antibiotics

**Antibiotics**	**ATC code**
Ceftazidime	J01DD02
Aztreonam	J01DF01
Piperacillin	J01CA12
Piperacillin and enzyme inhibitor	J01CR05
Tazobactam	J01CG02
Ticarcillin	J01CA13
Ticarcillin and enzyme inhibitor	J01CR03
Amoxicillin and enzyme inhibitor	J01CR02
Ticarcillin and enzyme inhibitor	J01CR03
Cefepime	J01DE01
Cefpirome	J01DE02
Imipenem and enzyme inhibitor	J01DH51
Meropenem	J01DH02
Doripenem	J01DH04
Colistin	J01XB01
Tigecycline	J01AA12
Vancomycin	J01XA01
Teicoplanin	J01XA02
Daptomycin	J01XX09

In models 1 to model 3, we adopted classification algorithms to develop an identification model. All criteria were converted into binary format. The criteria were (1) use of more than three types of antibiotics, (2) use of more than seven defined daily doses (DDD) of antibiotics, (3) use of more than 7DDD of cefazolin, (4) use of second-line antibiotics, (5) length of stay > 21 days, and (6) number of vessels obstructed > 2 (as a proxy indicator of duration of operation [18]). Although existing studies provided a suggestion base of criterion selection, such criteria varied across countries. For example, most surgeons in Taiwan prescribe 3 to 7 days of prophylactic antibiotic for CABG surgery, which is longer than the usual practice in western countries. Therefore, all criteria were further discussed with local surgeons. We defined a strict standard in model 1: the patient was identified as an SSI case only if all criteria were satisfied. The standard for model 2 was moderate: the patient was identified as an SSI case if more than three criteria were satisfied. The standard for model 3 was loose: the patient was identified as an SSI case if any of the criteria were satisfied.

We conducted a multivariable regression model in model 4 and used logistic regression to calculate the risk probability of cases with the same criteria we used in the classification algorithms. Different from models 1, 2, and 3, all binary variables were converted into continuous variables. The stepwise variable selection procedure was performed to avoid multicollinearity. We identified the optimal cut-off value from the ROC curves. The best cut-off value that corresponds to this perfect scenario (100% sensitivity and 100% specificity) would be at the upper left corner. In practice however, fewer tests are perfect, and one has to strike a balance between sensitivity and specificity. The Youden index was used to determine the optimal cut-off point [19].

The decision tree model was applied in model 5. This model is also known as the classification and regression tree (CART) model. Growing, stopping, and pruning of the tree were determined by Gini improvement measures [20,21]. We also adopted cross-validation to verify the final model. The model-building set was then used to establish a tree that was pruned by use of the validation set to achieve an estimation of the most appropriate tree through minimal cost-complexity pruning [20,21]. The criteria were the same as those for model 4.

### Model development, verification, and analysis of performance

The claims and surveillance data from medical center A were used to develop the alternative identification models with the use of IBM PASW 18 software, and the data from medical center B were used for verification. The healthcare-associated infection surveillance data were used as the reference standard.

Infection cases based on surveillance were identified by infection control personnel if the patient met the Taiwan CDC’s criterions, which are the same as that of the U.S. CDC. They manually review medical records of all patients at risk for the specified healthcare-associated infection.

The performance of our identification models was then analyzed using sensitivity (number of infected patients with positive test/total number of infected patients), specificity (number of non-infected patients with negative test/total number of non-infected patients), positive predictive value (number of infected patients with positive test/total number of patients with positive test), negative predictive value (number of non-infected patients with negative test/total number of patients with negative test), and accuracy (number of infected patients with positive test + number of non-infected patients with negative test/ total number of patients).

## Results

During the period 2005 to 2008, 1,017 patients underwent CABG surgery in medical center A (see Table 2), and 24 of them were confirmed as SSI cases by infection control professionals. The majority of patients were men (78.2%), and the mean age of the patients was 65 years. The mean level of number of vessels obstructed and length of stay were 1.88 and 18.09 days respectively. The mean number of types and doses of antibiotics were 1.57 and 8.27 respectively. Cefazolin was administered to 998 (97.15%) patients during the period of inpatient stay, and the mean number of doses was 3.93DDD. Second-line antibiotics were administered to 115 (11.58%) patients. The results also revealed that 45 cases of CABG SSI were identified by the ICD-9-CM approach, which represented an overestimation of the SSI rate among CABG cases in this medical center by the ICD-9-CM approach. The data also showed statistically significant differences between SSI cases and non-SSI cases for all variables except for age.

**Table 2:** Characteristics and medical use of CABG patients in medical center A during 2005-2008

	**All**	**Non-SSI**	**SSI**	**P-value**
N^‡^	1017	993 (97.64)	24 (2.36)	
Male^‡^	795 (78.2%)	781 (78.65)	14 (58.33)	0.017
Age (years)^†^	65.03 (10.86)	64.98 (10.86)	67.14 (10.95)	0.949
Number of vessels obstructed^†^	1.88 (0.34)	1.89 (0.33)	1.67 (0.56)	<0.001
Length of stay (days)^†^	18.09 (12.64)	17.11 (6.60)	58.29 (58.72)	<0.001
Length of stay (days)^*^	16 (8)	16 (7)	47.5 (21.5)	
Type of antibiotics^†^	1.57 (1.00)	1.51 (0.90)	3.71 (2.10)	<0.001
Doses of antibiotics^†^	8.27 (9.05)	7.89 (8.43)	24.07 (16.84)	<0.001
Use of cefazolin^‡^	998 (97.15)	975 (98.19)	13 (54.17)	<0.001
Doses of cefazolin^†^	3.93 (2.18)	3.96 (2.12)	2.65 (3.61)	<0.001
ICD-9-CM SSI code^‡^	45 (4.4%)	36 (3.63)	9 (37.50)	<0.001
Use of second-line antibiotics^‡^	115 (11.58)	115 (11.58)	20 (83.33)	<0.001

CABG: coronary artery bypass graft; SSI: surgical site infection.

^†^Mean(S.D) ^‡^N(%) ^*^Median(IQR).

**Figure 1:**
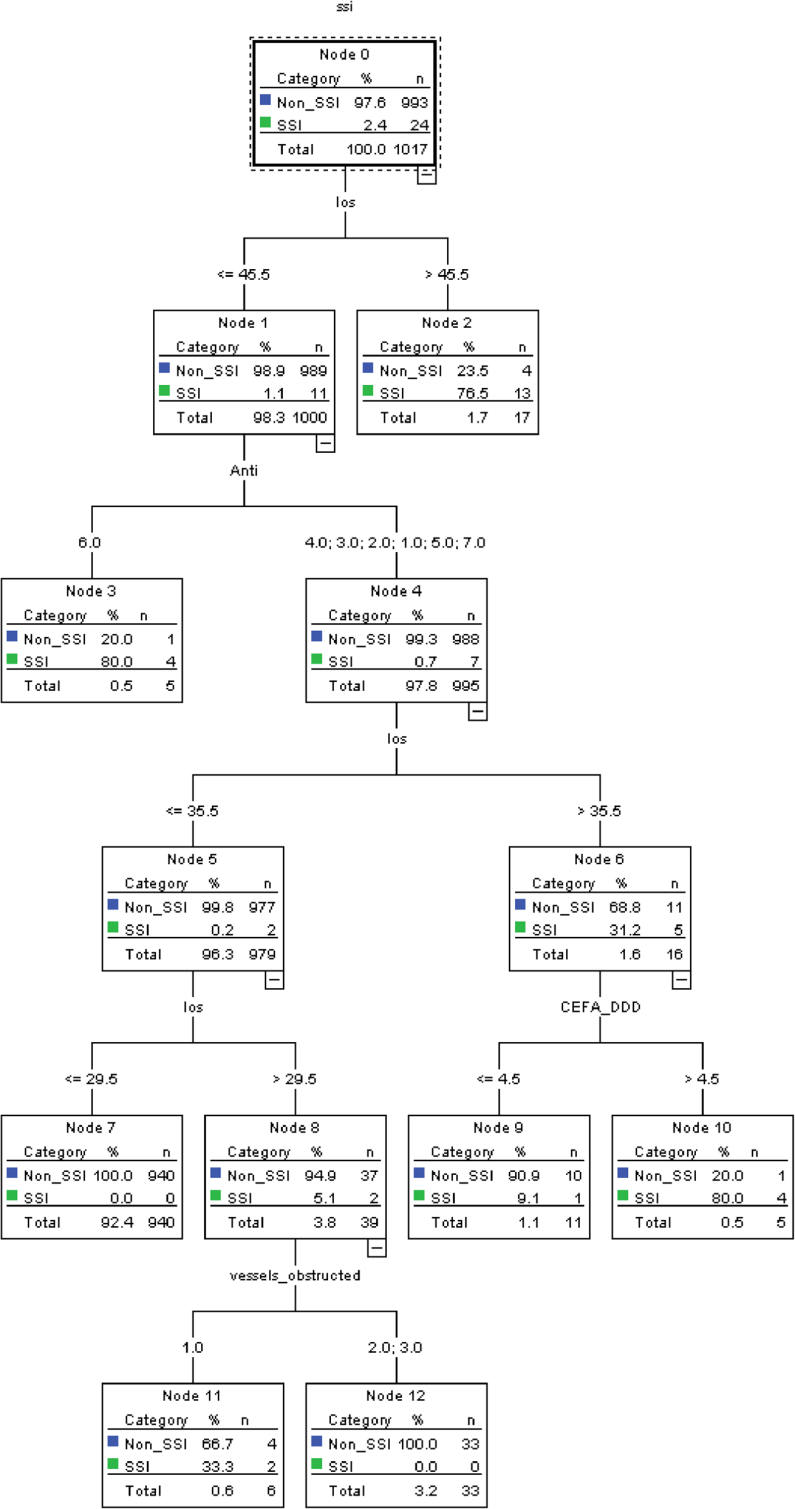
**Decision tree model for identifying cases of CABG surgical site infection.** SSI: surgical site infection; los: length of stay; Anti: type of antibiotics; CEFA_DDD:dosage of cefazolin; vessels_obstructed: number of vessels obstructed.

**Figure 2:**
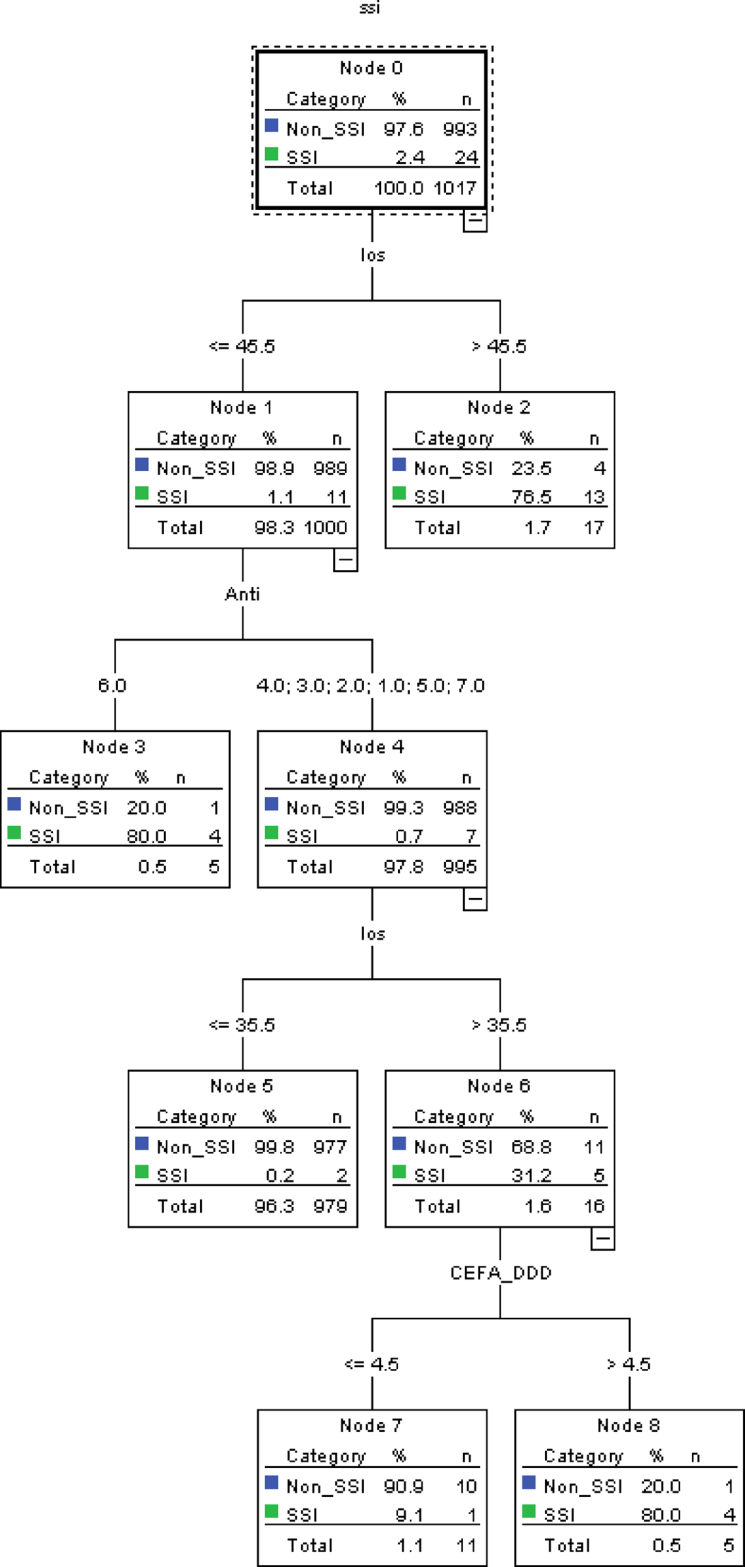
**Decision tree model for identifying cases of CABG surgical site infection (after pruning).** SSI: surgical site infection; los: length of stay; Anti: type of antibiotics; CEFA_DDD:dosage of cefazolin.

Table 3 demonstrates the results of stepwise selection in logistic regression model (training data), and only length of stay and type of antibiotics were retained in the model. The value of area under ROC curve was 0.989 (95% confidence interval: 0.983-0.996). According to the Youden index, the optimal cut-off value was 0.03. The value of area under ROC curve in the verification model was 0.978 (95% confidence interval: 0.961-0.994)Figure 1 is an illustration of the decision tree for identifying CABG surgical site infection, in which the most appropriate tree level is divided into seven terminal nodes. After model development by PASW 18, length of stay, type of antibiotics, dose of cefazolin, and number of vessels obstructed were retained in the model. Terminal node 3 indicates that when the length of stay is less than 45.5 days and the type of antibiotics is six, 80% of patients were identified as surgical site infection cases. The other combinatorial paths, interrelationships, predicted probabilities and populations are also illustrated in Figure 1. The tree diagram after pruning is presented in Figure 2.

**Table 3:** The results of stepwise selection of model 4-logistic regression model (training data)

**Parameter**	**Estimate**	**Standard error**	**p-value**
Intercept	10.0204	1.1212	<.0001
Length of stay	-0.1581	0.0233	<.0001
Type of antibiotics	-0.7427	0.1939	0.0001

Table 4 shows the performance of the training data (medical center A). The ICD-9-CM-based model had good specificity and negative predictive value, but its sensitivity and positive predictive value were poor. We found that performance varied in the other models except for negative predictive value. The performance of model 5 (without pruning) was excellent compared with that of the other models, especially in terms of positive predictive value. The performance of pruning model was the same with that of without pruning model.

**Table 4:** Performance of the ICD-9-CM-based and alternative models for identifying CABG SSIs (training data: medical center A)

	**Sensitivity**	**Specificity**	**PPV**	**NPV**	**Accuracy**
ICD-9-CM-based model	37.50% (9/24)	96.27% (956/993)	19.57% (9/46)	98.46% (956/971)	94.89% (965/1017)
Model 1	4.17% (1/24)	99.90% (992/993)	50.00% (1/2)	97.73% (992/1015)	97.64% (993/1017)
Model 2	54.17% (13/24)	96.78% (961/993)	28.89% (13/45)	98.87% (961/972)	95.77% (974/1017)
Model 3	100.00% (24/24)	3.22% (32/993)	2.44% (24/985)	100.00% (32/32)	5.51% (56/1017)
Model 4	100.00% (24/24)	94.56 (939/993)	30.77% (24/78)	100.00% (939/939)	94.69% (963/1017)
Model 5	87.50% (21/24)	99.40% (987/993)	77.78% (21/27)	99.70% (987/990)	99.12% (1008/1017)

% (numerator/denominator).

ICD-9-CM: The International Classification of Diseases, Ninth Revision, Clinical Modification; PPV: positive predictive value; NPV: negative predictive value. Model 1: the classification algorithms (strict); Model 2: the classification algorithms (moderate); Model 3: the classification algorithms (loose); Model 4: the multivariable regression model: Model 5: data mining-decision tree model.

We used the data of medical center B for model verification (Table 5). The performance of the verification models was quite similar to that of the training data. The ICD-9-CM-based model showed good specificity and negative predictive value, but the sensitivity and positive predictive value remained poor. We also found the performance of the other models to vary except for negative predictive value. As with the training data, the performance of model 5 was excellent compared with the other models, especially for positive predictive value.

**Table 5:** Performance of model verification for identifying CABG SSIs (verification data: medical center B)

	**Sensitivity**	**Specificity**	**PPV**	**NPV**	**Accuracy**
ICD-9-CM-based model	35.29 (6/17)	96.98 (803/828)	19.35 (6/31)	98.65 (803/814)	95.74 (809/845)
Model 1	5.88 (1/17)	99.76 (826/828)	33.33 (1/3)	98.10 (826/842)	97.87 (827/845)
Model 2	52.94 (9/17)	97.46 (807/828)	30.00 (9/30)	99.02 (807/815)	96.57 (816/845)
Model 3	100.00 (17/17)	2.42 (20/828)	2.06 (17/825)	100.00 (20/20)	4.38 (37/845)
Model 4	94.12 (16/17)	94.93 (786/828)	27.59 (16/58)	99.87 (786/787)	94.91 (802/845)
Model 5	88.24 (15/17)	99.28 (822/828)	71.43 (15/21)	99.76 (822/824)	99.05 (838/845)

% (numerator/denominator).

ICD-9-CM: The International Classification of Diseases, Ninth Revision, Clinical Modification; PPV: positive predictive value; NPV: negative predictive value. Model 1: the classification algorithms (strict); Model 2: the classification algorithms (moderate); Model 3: the classification algorithms (loose); Model 4: the multivariable regression model: Model 5: data mining-decision tree model.

## Discussion

The purpose of this study was to adopt three approaches to develop alternative models based on surrogate indicators to identify cases of CABG surgical site infection and to compare the performance among these models and the ICD-9-CM-based model. The main finding of this study was that the decision tree model we developed (model 5) offered better performance than that of the other identification models or the ICD-9-CM-based model, especially with regard to positive predictive value. The decision tree model was a decidedly better tool for identifying cases of SSI in the Taiwan National Health Insurance database. Furthermore, the results of this study could provide healthcare authorities with a tool to monitor surgical site infection among CABG patients, and can serve as a base for future research.

In Taiwan, surgeons prescribe antibiotics pre-, during and post-operation for preventing surgical site infection, and cefazolin is the most common one for prophylaxis use in CABG. However, there was no compulsory regulation on prescription, and surgeons can freely decide the type and dosage of antibiotics. Nevertheless, most surgeons usually prescribe first-line antibiotics (e.g. cefazolin) as prophylactic antibiotic.

This study highlighted three issues that are worthy of discussion. First, could surrogate markers be better than ICD-9-CM codes to identify cases of SSI in claims data? Researchers usually identified such cases through ICD-9-CM codes in claims data; however, many researchers have stated that use of ICD-9-CM codes to identify infection cases might be problematic [9,22,23]. In this study, infection rate calculated using ICD-9 CM codes was overestimated by factors of 2 and 2.2 respectively, in comparison with the actual rates in medical centers A and B. Previous studies adopted medical use as the surrogate marker for identifying cases of infection in claims data [1,4,24-27], and found better performance than that with the ICD-9-CM-based model. For example, Lee and colleagues [4] developed an alternative model based on antibiotic use to identify healthcare-associated infections in gastrectomy cases in Japan. Unlike their study that could differentiate the use of antibiotics between treatment and prophylaxis purposes, items recorded in the Taiwan National Health Insurance database are aggregated and were without time-relevant information. The present study could not identify the sequence of treatment, nor could it distinguish the purpose of use of antibiotics. Furthermore, the current study was inspired by Lee et al., hence we selected “more than three types of antibiotics and use of second-line antibiotics” as the criterion. The results that the performance of all models except for model 2 were better than or equal to that of the ICD-9-CM-based model implied that medical use could be a surrogate for ICD-9-CM codes in claims data.

Second, positive predictive value should be the criterion to determine the identification model. Most studies used sensitivity and specificity as indicators to evaluate the performance. The results of other studies [1,4,17] which used positive predictive value to evaluate the performance showed that the performance of positive predictive value was not good because of the lack of infection events [5,28]. For example, in Lee et al., study [4], sensitivity, specificity, and negative predictive values were over 90%, but the positive predictive value was around 70%.Similar with previous studies, current study found that the positive predictive value was lower than the other indicators, except for model 1. Besides, the positive predictive value of model 5 was significantly better than the others.

Third, the decision tree model should be the recommended approach. In general, classification algorithms were intuitional and this method was easy to perform. However, determination of the optimal criteria and definition of the cutoff point for each criterion (e.g., length of stay >7 days) were challenges to work out. Although a multivariable regression model can consider all variables simultaneously, which can also deal with continuous variables, it has limitations in dealing with high-dimension variables [21,29]. And the performances of the decision tree model were better than others, which can be attributed to the ability of the decision tree model to classify high-dimensional data [29]. The decision tree model is a computer-aided decision-making approach that can help researchers figure out the optimal solution to find cases that they are concerned about. However, it is not widely used in academic medical societies, and most colleagues in medical societies might not be familiar with this approach. As shown in the results, the performances were similar between Model 4 and Model 5, except for the positive predictive value. Furthermore, the current study found that while the sensitivity of model 4 was equal to model 5 (87.5%), the specificity, positive predictive value, negative predictive value and accuracy were 97.78% (971/993), 48.84% (21/43), 99.69% (971/974), and 97.5% (992/1017) respectively. The results gained demonstrated the better performance of model 5 over model 4.

This study has several limitations. Firstly, there is room for improvement of the positive predictive value (>75%) of Model 5, and its performance was not worse than that reported in existing studies [1,4,17]. However, we could not distinguish between therapeutic and prophylactic use of antibiotics because of a limitation of the dataset. If we could obtain such information, it might be useful in improving performance. Secondly, we used six criteria, and we could only select loose, moderate, and strict combinations of these criteria to develop identification models that represent the performance of all possible combinations. Thirdly, study generalizability, identification model development, and model verification were limited by the use of the National Health Insurance claims data and surveillance data from two medical centers. Theoretically, the findings of this study could be applied to other levels of hospitals in Taiwan as well as other countries and payment systems. The major reason is the methodological strength of the decision tree model. Nevertheless, this merits further examination, as we do not have data from different hospital levels and different countries. Fourthly, the reference standard in the current study was the healthcare-associated infection surveillance data.Infection cases were manually identified by infection control professionals, and Taiwan CDC provided training and explicit criteria. However, in some special cases, inconsistencies might exist among hospitals, although such a situation was rare [1]. Furthermore, some healthcare-associated infection cases occurred after discharge. If patients did not return to the same hospital, they would not be identified as healthcare-associated infection cases in the surveillance data [12].

## Conclusion

In summary, use of ICD-9-CM codes with National Health Insurance claims data in Taiwan to identify cases of CABG SSI might overestimate the number of cases by a factor of two. However, the accuracy of the decision tree model, with medical uses as its parameters, was better than that of the ICD-9-CM-based model, especially in the sensitivity and positive predictive value. Use of the decision tree model to identify cases of CABG surgical site infection can improve the accuracy of patient-level outcome research. This model should be considered in future research using claims data.

## Competing interests

The authors declare that they have no competing interests.

## Authors’ contributions

THY developed study concept, analyzed the data and drafted the manuscript, YCH was involved in collecting data and drafting the manuscript, KCL reviewed the methods and results and revised manuscript, and KPC collected data, coordinated the study, and revised manuscript. All of the authors have prepared, read, and approved the manuscript.

## Pre-publication history

The pre-publication history for this paper can be accessed here:

http://www.biomedcentral.com/1472-6947/14/42/prepub
